# Characteristics of Microbial Communities of *Pachygrontha antennata* (Hemiptera: Pachygronthidae) in Relation to Habitat Variables

**DOI:** 10.3390/ijerph16234668

**Published:** 2019-11-23

**Authors:** Jae-Yeon Kang, Yong-Su Kwon, Gilsang Jeong, Injung An, Soyeon Park

**Affiliations:** 1 Evolutionary Ecology Research Team, National Institute of Ecology, Seocheon-gun 33657, Korea; jaeyoni0809@gmail.com (J.-Y.K.); injungg0917@nie.re.kr (I.A.); 2 EcoBank Team, National Institute of Ecology, Seocheon-gun 33657, Korea; kwonys@nie.re.kr; 3 Long term Ecological Research Team, National Institute of Ecology, Seocheon-gun 33657, Korea; gilsangj@nie.re.kr

**Keywords:** pachygronthidae, *pachygrontha antennata*, microbial community, land use, habitat variable

## Abstract

The microbial community interacts with the environment and the health and immune function of its host both directly and indirectly. However, very few studies about microbial communities have considered habitat and external environmental variables. This study examined environmental influences on the microbial community of *Pachygrontha antennata*, which is found in various habitats (e.g., urban, forested, and agricultural areas). The results demonstrated that the composition of the microbial community differed according to land use, while the bacterial diversity did not. In urban areas with high environmental heterogeneity, microbial community diversity tended to be high. Furthermore, bacteria in forests and agricultural areas (e.g., *Paraburkholderia*, *Burkholderia*) have been found to be highly correlated with habitat variables. Therefore, we suggest that habitat variables should be considered in future symbiotic studies.

## 1. Introduction

A microbial community refers to all of the intracellular and extracellular bacteria that exist within a single organism. Microbial communities differentiate into ecological niches in the host organism within a limited space to relieve unnecessary interspecies competition, contributing to biodiversity and playing an important role in host adaptation and fitness [1,2,3,4,5]. Bacteria can be both horizontally and vertically transmitted. Vertical transmission refers to transmission from a mother to an offspring, as in the case of mitochondria, whereas horizontal transmission refers to transmission from the external environment. Environmentally derived bacteria can be spread via horizontal transmissions between organisms, and the composition and diversity of these bacteria may vary according to environmental variables such as habitat, diet, and social contact [5,6].

Varying landscapes comprise diverse environmental variables, such as climate, resources, and organisms, which are directly associated with microbial foraging activities and interspecies interactions. The diversity in a given landscape causes a uniquely adapted complex network of microbial interactions to arise [7]. For example, ladybugs are found in highly diverse habitats (e.g., soybean fields and prairies), and their microbial community compositions differ depending on the habitat in which they are found [7]. The microbial community of the house sparrow (*Passer domesticus*) is also more diverse in agricultural areas than in urban areas [8]. Urbanization and subsequent habitat fragmentation have recently increased environmental heterogeneity, including within the same urban habitat, making it difficult to make accurate biological and environmental predictions, such as those regarding the invasion of exotic species, unpredictable climate, and development of heat islands [9]. To improve prediction capabilities, a better understanding of biological responses with reference to the landscape and actual environmental data is necessary.

Insects are the largest group taxonomically classified, and exhibit highly diverse feeding habits. They widely adapted to most environments. The guts of insects are known to contain enterobacteria that produce various bioactive substances, such as digestive enzymes, and dissolve contaminants from external sources [10]. Insects within Hemiptera are sensitive to vegetation structure [11,12,13,14,15,16] and contamination [17]. However, very few studies have considered habitat variables when characterizing microbial communities of insects, including the Hemiptera.

*Pachygrontha antennata* [18] belongs to the order Hemiptera and family Pachygronthidae, and this species is a major pest in agricultural areas. The adult insects are typically found between April and October, and are distributed across Korea, Japan, China, and Russia [19]. This insect was suitable for our examination of the environmental influences on the microbial community for two reasons. First, *P. antennata* is a generalist and it commonly inhabits the areas that we investigated. Thus, comparisons could be made for each land use, and natural experiments assessing the environmental influence on microbial communities could be performed. Second, the use of this insect in our study minimized the effects of intracellular bacteria. *Wolbachia*, which are maternally inherited intracellular bacteria, have been shown to alter the relative abundance of microbial communities in host insects [20]. Accordingly, to assess the differences in the microbial community due to environmental influences, host insects should not be infected with *Wolbachia*. Thus, all *P. antennata* used in this study were *Wolbachia*-free.

Despite the importance of microbial communities for host fitness, few studies have investigated the characteristics of host-insect-associated microbial communities. Furthermore, due to the lack of empirical studies on the bacteria associated with the environmental variables, it is challenging to predict the dynamics of the microbial communities given an environmental change. Notably, *Burkholderia* are the only bacteria that have been identified in *P. antennata* [21]. We assessed the effect of environmental influences on the microbial community of *P. antennata*. In particular, the effects on the microbial community caused by the macrohabitat and microhabitat variables as environmental variables were examined. Previous studies have primarily analyzed the impact of land use using GIS (Geographical Information System) data [7,8,22]. Here, we considered not only the GIS data, but also data pertaining to the actual environment (ambient temperature and degree of coverage) that the host insect is faced with. Thus, we investigated (1) any variance in the structure (diversity and composition) of the microbial community of *P. antennata* based on habitat variables, and (2) any associations between the microbial community and varying habitat variables. The evaluation of microbial communities of insects in association with the environmental variables can be exploited to control agricultural pests and disease-transmitting insects, as well as to conserve endangered insects [23,24,25].

## 2. Materials and Methods

### 2.1. Microbial Community

To compare the compositions of the microbial community by habitat type, 18 samples of *P. antennata* were collected using the sweeping method at eight sites in five regions in Korea (Seoul, Daejeon, Buyeo, Seocheon, and Gunsan) during the summer of 2018. Table 1 lists detailed information about the collection sites, including GPS coordinates. There was no geographic isolation, such as mountain ranges, between the five regions where samples were collected. The collected samples were immediately placed in 100% ethanol (EtOH) and stored at −20 °C until genomic DNA could be extracted. Samples were vortexed twice in 100% EtOH to remove foreign contaminants. After samples were frozen with liquid nitrogen, they were lysed using two 3 mm beads and a tissue lyser (30 rpm/s, 20 s) [26,27]. Genomic DNA (gDNA) was extracted from the lysed samples using the DNeasy Blood & Tissue kit (Qiagen, Hilden, Germany).

To construct a library from the extracted gDNA, the V3 and V4 regions of the 16S rRNA gene were amplified. The PCR primer set used during the preparation was comprised of an adaptor sequence and a target sequence [28]. The Illumina MiSeq platform was used to obtain nucleotide sequences of microbial communities. A large amount of nucleotide sequence information was obtained from the prepared gDNA library. Raw sequences were trimmed to remove the primer sequence and other unnecessary sequences (i.e., low quality amplicons, non-target amplicons, and chimeric amplicons). After trimming, species-level identification with 97% 16S similarity was conducted using the EzBiocloud database (database version: PKSSU4.0) [29]. The metagenome data were also normalized for 16S gene copy number and read counts [30], and the alpha diversity indices (Shannon index and phylogenetic diversity) were calculated using the EzBiocloud software.

### 2.2. Habitat Variables

Thirteen habitat variables which could influence the composition of the microbial community in *P. antennata* were used in our analysis, including microhabitat variables (ambient temperature and cover degree) and macrohabitat variables (land use) (Table 2). Temperature was measured hourly at each location using a temperature logger (EL-USB-2-LCD+, LASCAR Instrument). The loggers were installed within a 30 m radius of the representative sites of insect collection at a height of about 2.5 m, out of direct sunlight. The cover degree was measured within a 10 × 10 m area of the sampling site using the ocular method in the FIA (Forest Inventory and Analysis) field manual (ver. 3.0) for estimating cover degree [31].

Land use data were obtained from the Ministry of Environment, Korea. Land use data were extracted for a 500 m radius of each collection site [16,32,33] using GIS software (version 10.1, ArcGIS, Redlands, CA, United States), and these data were used to calculate the proportion (%) of each land use. To avoid issues with GIS, distances that covered the largest area of a particular region were used, and the GPS coordinates were used as the site of origin to minimize potential errors caused by the GIS process.

### 2.3. Data Analysis

#### 2.3.1. Multivariate Analyses

The differences in macrohabitat type of the microbial communities of *P. antennata* were analyzed in relation to external habitat variables using multivariate statistical analyses such as cluster analysis and principal component analysis (PCA). Cluster analysis was performed to classify the differences in macrohabitat type of eight sampling sites based on the proportion of land use (urban, agricultural, forest, grassland, bare land, and watershed area) using Ward’s linkage method with a Euclidean distance measure. A multi-response permutation procedure (MRPP) analysis was performed on the classified clusters to verify their significance [34]. Principal component analysis was conducted to characterize spatial patterns of microbial communities of *P. antennata* in relation to habitat variables. During the data preprocessing procedure, we applied cut-off filters of >0.1% and >1% based on abundance level, and these cut-offs were used to select the predominant taxa [35]. The abundance of the microbial community of each sample underwent a natural-logarithm transformation (ln (x + 1)) to reduce variation and to meet the assumption of normality. After transformation, each sample was rescaled to between 0 and 1 with min-max transformation to normalize all taxa for the comparison analysis. The cluster analysis, MRPP, and PCA were performed with PC-ORD 5.0 (Wild Blueberry Media LLC, Oregon, OR, United States) [36].

#### 2.3.2. Statistical Analyses

Spearman rank correlation analysis was conducted to assess the correlation between macrohabitat and microhabitat variables. A Dunn’s multiple-comparisons test after a Kruskal-Wallis test (K-W test) was used to compare the differences in habitat variables among the clusters. These analyses were conducted using STATISTICA software (version 7, StatSoft, Inc., Arizona, CA, USA). The Wilcoxon rank-sum test was also used to compare the alpha diversity of the microbial community and the relative abundance (%) of particular bacteria at the genus level based on each habitat type divided by the cluster analysis. This analysis was conducted using the R software (version 3.6.1, R Development Core Team, Auckland, New Zealand).

## 3. Results

### 3.1. Characteristics of Macro and Microhabitat Variables

Correlations between the land use proportion and cover degree as well as ambient temperatures at sampling sites are shown in Table 3. The proportion of urban area was positively correlated with minimum temperature (Temp_min; *r* = 0.72, *p* < 0.01) and arbor (*r* = 0.57, *p* < 0.01). Canopy (*r* = −0.85, *p* < 0.01), maximum temperature (Temp_max; *r* = 0.48, *p* < 0.05), and cover degree, such as grass (*r* = 0.51, *p* < 0.05), arbor (*r* = −0.48, *p* < 0.05), and shrub (*r* = −0.50, *p* < 0.05), were significantly correlated with agricultural area. Forest area had significant negative correlations with all ambient temperature factors (*p* < 0.01). Meanwhile, grassland and bare land were not significantly correlated with any microhabitat variables (*p* > 0.05).

### 3.2. Classification of Macrohabitat Type

**Figure 1:**
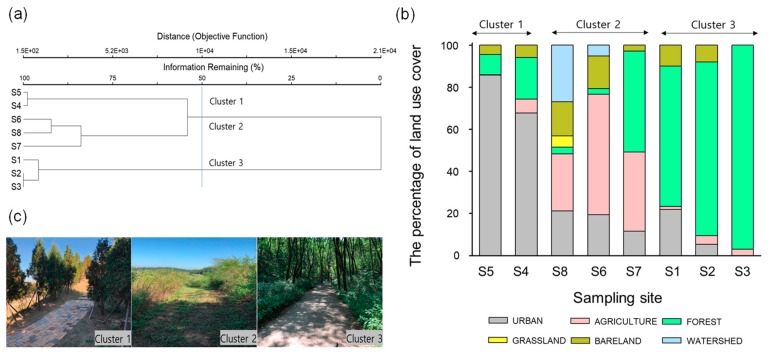
(**a**) Classification of eight sampling sites based on the composition of land use through a cluster analysis with Ward’s linkage method using a Euclidean distance measure. (**b**) The percentage of land use in the sampling sites. (**c**) The representative images in each cluster defined by cluster analysis.

Cluster analysis was performed to classify the differences of the macrohabitat type of the eight sampling sites, which were categorized into three clusters (1–3) based on the similarity of land use (Figure 1a). The MRPP showed significant differences among clusters (A = 0.53, *p* = 0.0024). Among the eight sites, S5 and S4, which belonged to Cluster 1, had high urban ratios of 86% and 68%, respectively, while the other sites were less than 22% urban (Figure 1b). In addition, the forested areas accounted for more than 67% of the sites in Cluster 3, while the sites in Cluster 2 consisted of more than 27% agricultural areas.

According to our analysis, land use differed significantly among clusters (Table 4). Urban area was highest in Cluster 1 (76.7 ± 9.9%) compared to all other clusters (Dunn’ s test, *p* < 0.05; Table 3). Forested area was highest in Cluster 3 (77.0 ± 12.5%), whereas agricultural area was highest in Cluster 2 (45.7 ± 13.2%; Dunn’ s test, *p* < 0.05). Meanwhile, the proportions of grassland, bare land, and watershed area were not significantly different among clusters (K-W test, *p* > 0.05).

In addition, ambient temperature, except for maximum temperature and cover degree, was significantly different among clusters (K-W test, *p* < 0.05; Table 4). Cluster 1 showed high values for average temperature (Temp_ave) and minimum temperature (Temp_min), while Cluster 3 exhibited the lowest values (Dunn’ s test, *p* < 0.05). Meanwhile, maximum temperature (Temp_max) was relatively low in Cluster 3, although there was no significant difference between clusters (K-W test, *p* > 0.05). Finally, the proportion of arbors and shrubs was highest in Cluster 1, whereas the proportion of grass was highest in Cluster 2 and lowest in Cluster 1 (Dunn’ s test, *p* < 0.05).

### 3.3. Difference in Microbial Communities

To analyze the microbial communities, nucleotide sequences of an average length of 424 bp per individual were obtained from 18 samples of *P. antennata*. A total of 670 taxa were identified from the collected nucleotide sequences, including 27 predominant taxa (abundance level > 0.1%) and seven major predominant taxa (abundance level > 1%; Table 5). *Paraburkholderia* (48.1%) and *Caballeronia* (32.4%) were the most abundant taxa and made up 80.5% of the microbial community. However, *Caballeronia* (43.9%) was most abundant in Cluster 1, and *Paraburkholderia* (25.3%) was a sub-dominant taxon. Taxon richness was highest in Cluster 1 (427 taxa), followed by Cluster 3 (314 taxa) and Cluster 1 (171 taxa).

**Figure 2:**
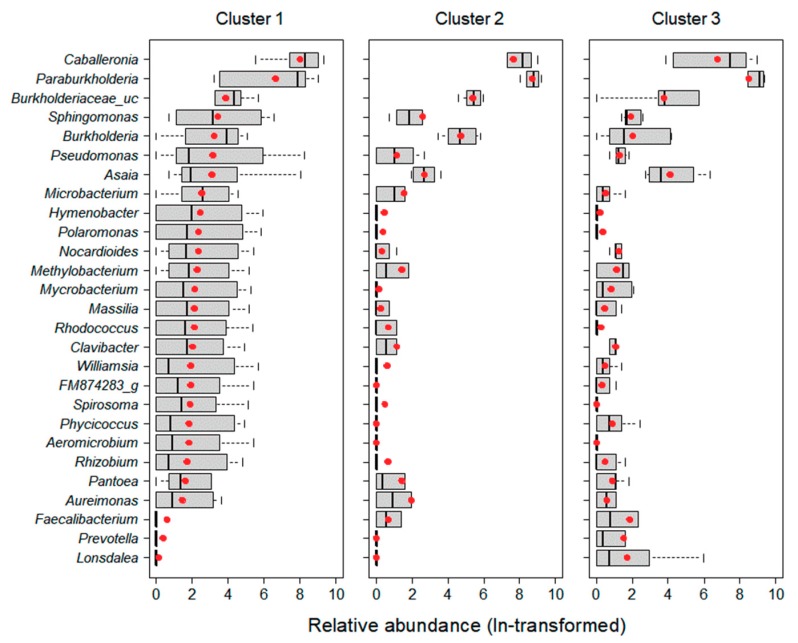
Rescaled abundance of 27 predominant taxa in three clusters by cluster analysis. Abundance was transformed by natural-logarithm (ln (x + 1)). The boxplots consist of a median (black line), mean (red circle), 25–75% percentiles, and non-outlier range (error bar).

The relative abundance of 27 predominant taxa in each cluster was transformed by natural-logarithm (Figure 2). Microbial taxa showing high relative abundance were different between clusters. The relative abundance of 19 taxa in Cluster 1 was higher than in other clusters. In Cluster 2, the relative abundances of *Burkholderiaceae_uc*, *Paraburkholderia*, *Burkholderia*, and *Aureimonas* were high, while in Cluster 3, the relative abundances of *Asaia*, *Faecalibacterium*, *Prevotella*, and *Lonsdalea* were high.

**Figure 3:**
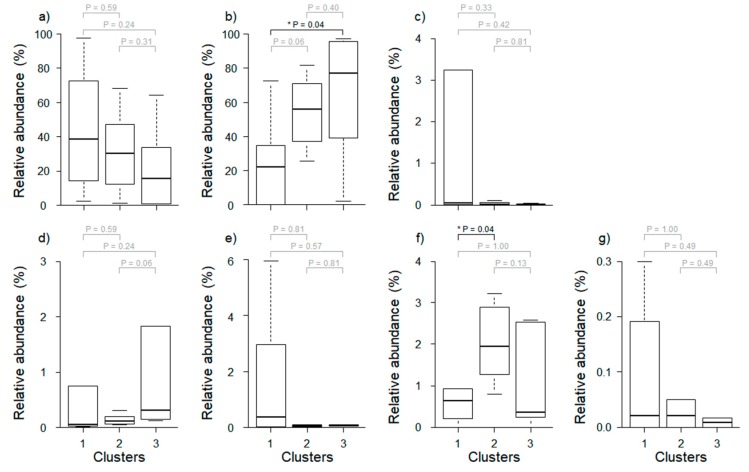
Comparison of relative abundance of major predominant taxa (cut-off filter based on abundance level > 1%) among three different clusters. (**a**) *Caballeronia*, (**b**) *Paraburkholderia*, (**c**) *Pseudomonas*, (**d**) *Asaia*, (**e**) *Sphingomonas*, (**f**) *Burkholderiaceae_uc*, and (**g**) *Aureimonas*. Asterisks represent significant differences based on the Wilcoxon rank sum test (* *p* < 0.05). Gray *p*-Values indicate no significant difference among clusters.

We evaluated the relative abundance of seven major predominant taxa (cut-off filter based on abundance level > 1%) among clusters defined by cluster analysis (Figure 3). Some predominant taxa such as *Paraburkholderia* and *Burkholderiaceae_uc* showed significant differences, but the other five predominant taxa did not. *Paraburkholderia* differed significantly between Clusters 1 and 3 (Wilcoxon rank-sum test, *p* = 0.04), and *Burkholderiaceae_uc* differed significantly between Clusters 1 and 2 (Wilcoxon rank-sum test, *p* = 0.04). Meanwhile, the relative abundance of *Pseudomonas* and *Sphingomonas* were higher in Cluster 1 than in other clusters, although the difference was not statistically significant (Wilcoxon rank-sum test, *p* > 0.05).

**Figure 4:**
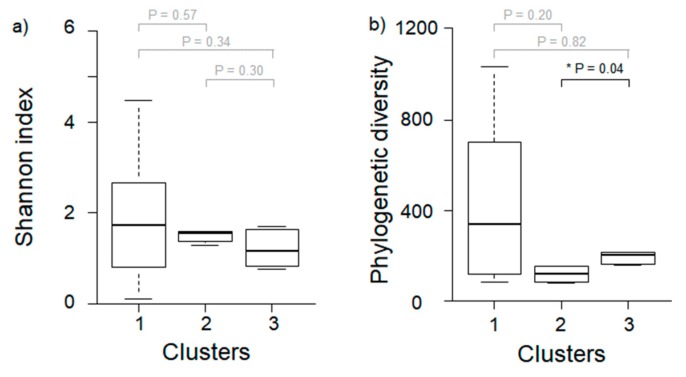
Difference in diversity of microbial community in *P. antennata* among three different clusters. (**a**) Shannon index, (**b**) phylogenetic diversity. Asterisks represent significant differences based on Wilcoxon rank-sum test (*p* < 0.05). Gray *p* values indicate no significant difference among clusters.

Based on results from the Wilcoxon rank-sum test, the phylogenetic diversity for the microbial community of *P. antennata* was significantly different between Clusters 2 and 3 (Wilcoxon rank-sum test, *p* = 0.04; Figure 4). Furthermore, the Shannon index was not significantly different among clusters (Wilcoxon rank-sum test, Cluster 1–2, *p* = 0.57; Cluster 1–3, *p* = 0.34; Cluster 2–3, *p* = 0.30). Raw sequence data generated for this study are available from NCBI Sequence Read Archive (SRA; BioProject PRJNA588651).

### 3.4. Ordination with Microbial Community

**Figure 5:**
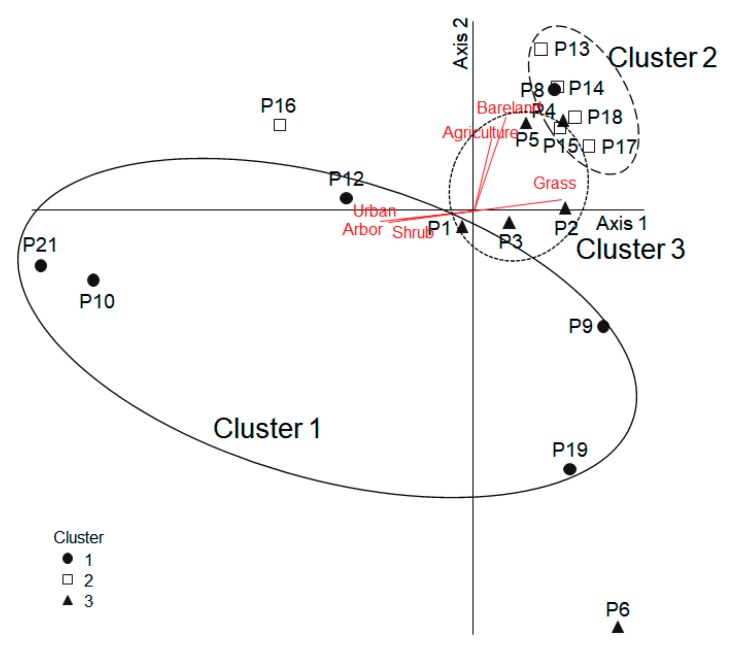
Principal component analysis ordination based on the differences in microbial community of *P. antennata*. Principal component analysis ordination based on the differences in relative abundance of microbial community in *P. antennata* (less than 0.1% were excluded). The environmental variables that showed significant correlation coefficients (*p* < 0.05) with the first two principal axes are shown as gray lines. The line length indicates the magnitude of the correlation and the line direction implies a negative or positive correlation with each axis.

The microbial communities of *P. antennata* could be ordinated on the biplot based on their contribution to the community ordination using PCA (Figure 5). The cumulative variances of Axes 1 (57.6%) and 2 (13.8%) was 67.4%. The three clusters divided by cluster analysis were subjected to PCA. The samples in Cluster 1 were mainly located on the lower left quadrant of the ordination map, and samples in Cluster 2 were principally in the upper right quadrant. Based on the correlation coefficients between environmental variables and PCA axis scores, the PCA axes were significantly correlated with some habitat variables, and were visualized on the PCA ordination map with vector length and direction. As a result, the urban area (*r* = −0.57, *p* < 0.05) and the proportion of arbor (*r* = −0.54, *p* < 0.05) and shrubs (*r* = −0.50, *p* < 0.05) were negatively correlated with Axis 1, whereas the proportion of grass (*r* = 0.55, *p* < 0.05) was positively correlated with Axis 1. Axis 2 was highly correlated with the bare land area and agriculture area (*r* = 0.57 and *r* = 0.50, respectively; *p* < 0.05).

## 4. Discussion

In our study, macrohabitat and microhabitat variables were significantly correlated (Table 3), and they represented the actual environment inhabited by *P. antennata*. We also noted some differences in the composition of the microbial communities of *P. antennata* according to the habitat variables. Although not statistically significant, there was a trend which suggested that these factors impacted the bacterial diversity at a relatively low level. Therefore, our findings suggested that the composition of microbial communities can vary depending on the habitat variables.

The composition of microbial communities showed distinct differences based on land use, especially for those grouped in Cluster 1, with the characteristics of urban areas, which were relatively abundant in 19 predominant taxa (Figure 2). Since habitat composition is directly linked to food sources for insect hosts, the diversity of microbial communities is likely to be richer in habitats with more diverse food sources [22]. Generally, urban areas have anthropogenic disturbances and high temperatures [37], which reduces the range of food sources [38] and subsequently decreases microbial diversity [22]. However, in some cases, urbanization may induce environmental heterogeneity [9,39]. Depending on the habitat compositions of urban areas, food source availability and diversity of bacteria may increase. For instance, the microbiome of male birds was found to be highly diverse in urban areas with a specific set of environmental conditions (tree/scrub/grass/impervious area), while in rural areas, the diversity of their microbiome was low due to considerably simpler habitats and cultivation practices [22]. These previously uncovered patterns are similar to our results. Specifically, although it was not significantly different, the Shannon index (1.92 ± 1.39) and phylogenetic diversity (436 ± 344) in Cluster 1, which had a high proportion of urban area, indicated greater diversity than at other sites (Figure 4). In particular, Cluster 1 was significantly higher than the other clusters at all cover degrees except grass, and it had relatively diverse environmental conditions. Taken together, urban heterogeneity appears to positively influence the diversity of microbial communities.

There was a marked difference in the composition of the microbial communities when analyzed from the perspective of land use (Figure 2). *Paraburkholderia* and *Caballeronia* were the predominant taxa in all clusters (Table 5), and the relative abundance of *Paraburkholderia* was significantly higher, especially in Cluster 3, which had a high proportion of forests (Wilcoxon rank-sum test, *p* = 0.04). *Paraburkholderia* promotes plant growth [40,41,42]. Because plants are typically more abundant in forests than in other habitats, the forest soil is likely enriched in *Paraburkholderia*.

*Burkholderia* is a major genus of bacteria found in various types of environments (soil, plants, insects, molds, and humans) [43]. The relative abundance of *Burkholderia* (1.3 ± 0.9%) and *Burkholderia_uc* (2.0 ± 0.9%) was highest in Cluster 2, which had a high proportion of agricultural area (Figure 3 and Figure 4). *Burkholderia* breaks down fenitrothion in the insect *Riptortus clavatus.* This relative abundance is similar to that observed by Kikuchi et al. [44] who confirmed the presence of *Burkholderia* spp. and demonstrated that these bacteria acquire resistance to the insecticide fenitrothion in agricultural areas and form a mutually beneficial symbiotic relationship. Since *Burkholderia* is transmitted horizontally, each generation can obtain these commensal bacteria from their environment. Based on these results, it is likely that *P. antennata* was subjected to selective pressure to develop an increased tolerance to fenitrothion.

Possible reasons for land use affecting microbial communities are as follows: first, the food source is a key factor in microbial communities and can be altered by different by land use [45]. Since *P. antennata* is not a specialist, it can consume different plants depending on where it lives. This may cause differences in microbial communities among the different types of land use. For example, *Aphis gossypii* show different densities of *Buchnera*, an obligate bacterium, depending on their host plants [46]. In addition, in the honeybee, the composition of the microbial community differs according to the forage type [47]. This has been explained by the fact that because of the different secondary metabolites present in plants, the amounts of bacteria that promote the decomposition of certain pollen components can change. Second, specific environmental conditions resulting from land use can affect the composition of microbial communities. For example, in agricultural areas containing pesticides, insecticide-degrading bacterial populations are enriched (e.g., *Burkholderia*) [44], and red weaver ants in urban areas harbor pathogenic bacteria [48]. Our results showed that *Pseudomonas*, a pathogenic bacterium [49], had the highest relative abundance in urban areas. Collectively, our findings highlight the possibility that different food sources and specific environmental conditions resulting from land use may influence the microbial community of *P. antennata*, and further comprehensive studies (e.g., laboratory and outdoor) are required for elucidating the causes behind these differences.

## 5. Conclusions

The results revealed that the compositions of the microbial communities of *P. antennata* differ based on habitat variables, although these factors have a relatively small impact on bacterial diversity. In urban areas with high environmental heterogeneity, the diversity of microbial communities tended to be high. In forests and agricultural areas, bacteria (*Paraburkholderia, Burkholderia*) that were highly correlated to habitat variableswere also identified. Our findings suggest that the structure of microbial communities can vary depending on land use or environmental variables. Furthermore, we demonstrated that human activities affect habitat compositions as well as environmental variables, insect activities, and microbial communities. Therefore, differences in microbial communities stemming from environmental conditions should be considered not only when assessing the characteristics of symbionts, but also for the conservation of host animals.

## Figures and Tables

**Table 1:** Location of sampling sites for collecting *P. antennata*.

Site	Region	Coordination		Samples
		Latitude	Longitude	
S1	Daejeon	36.37441	127.3486	P1, P2, P3
S2	Daejeon	36.11464	127.3297	P4, P5
S3	Daejeon	36.30056	126.9169	P6
S4	Buyeo	36.28120	126.9169	P8, P9, P19
S5	Seoul	37.51008	127.0767	P10, P12, P21
S6	Buyeo	36.26959	126.9122	P13, P14, P15
S7	Seocheon	36.02857	126.7268	P16, P17
S8	Gunsan	36.00767	126.7569	P18

**Table 2:** Description of micro and macrohabitat variables at the eight sampling sites.

Category		Variables	Abbreviation	Mean (± SD)
Microhabitat variable (10 × 10 m)	Ambient temperature	Average daily temperature (°C)	Temp_ave	24.8 ± 2.2
		Maximum daily temperature (°C)	Temp_max	32.0 ± 3.6
		Minimum daily temperature (°C)	Temp_min	18.7 ± 1.7
	Cover degree	Grass (%)	Grass	59.1 ± 36.1
		Arbor (%)	Arbor	19.7 ± 18.7
		Shrub (%)	Shrub	21.2 ± 19.5
		Canopy (%)	Canopy	27.8 ± 18.8
Macrohabitat variable (500 m radius)	Land use	Urban area (%)	Urban	35.6 ± 31.1
		Agriculture area (%)	Agriculture	17.2 ± 22.0
		Forest area (%)	Forest	36.5 ± 32.9
		Grassland (%)	Grassland	0.3 ± 1.3
		Bare land (%)	Bareland	8.0 ± 4.9
		Watershed area (%)	Watershed	2.4 ± 6.4

**Table 3:** Spearman rank correlation coefficients between microhabitat and macrohabitat variables at eight sampling sites (** *p* < 0.01, * *p* < 0.05).

Land Use	Cover Degree				Ambient Temperature		
	Grass	Arbor	Shrub	Canopy	Temp_ave	Temp_max	Temp_min
Urban	−0.43	0.57 *	0.25	0.37	0.46	0.27	0.72 **
Agriculture	0.51 *	−0.48 *	−0.50 *	−0.85 **	0.30	0.48 *	−0.19
Forest	0.07	−0.16	0.06	0.35	−0.66 **	−0.81 **	−0.63 **
Grassland	0.31	−0.31	−0.31	−0.36	−0.02	0.12	0.12
Bare land	0.27	−0.30	−0.28	−0.17	0.21	0.45	−0.05
Watershed	0.31	−0.31	−0.31	−0.56 *	0.32	0.63 **	0.11

**Table 4:** Differences of macro and microhabitat variables among the three clusters defined by the cluster analysis. The number indicates the mean (± SD) in each cluster. Different lowercase letters (a and b) in the table indicate significant differences based on Dunn’s test (*p* < 0.05). Habitat variable acronyms are given in Table 2.

Category		Variables	Clusters		
			1	2	3
Macrohabitat variables	Land use	Urban	76.7 ± 9.9 ^a^	17.2 ± 4.3 ^b^	12.8 ± 10.2 ^b^
		Agriculture	3.5 ± 3.6 ^b^	45.7 ± 13.2 ^a^	2.5 ± 1.4 ^b^
		Forest	14.7 ± 5.5 ^b^	17.9 ± 23.3 ^b^	77.0 ± 12.5 ^a^
		Grassland	0.0 ± 0.0	0.9 ± 2.2	0.0 ± 0.0
		Bareland	5.1 ± 0.8	11.3 ± 6.6	7.7 ± 3.9
		Watershed	0.0 ± 0.0	7.1 ± 10.0	0.0 ± 0.0
Microhabitat variables	Ambient temperature	Temp_ave	26.7 ± 0.7 ^a^	25.2 ± 2.2 ^ab^	22.5 ± 0.1 ^b^
		Temp_max	33.4 ± 2.5	33.8 ± 4.6	28.9 ± 0.2
		Temp_min	20.7 ± 0.4 ^a^	18.4 ± 1.2 ^ab^	17.1 ± 0.3 ^b^
	Cover degree	Grass	26.7 ± 29.2 ^b^	89.1 ± 6.6 ^a^	61.7 ± 34.3 ^ab^
		Arbor	41.7 ± 9.1 ^a^	3.5 ± 2.8 ^b^	13.8 ± 12.6 ^b^
		Shrub	31.7 ± 20.1 ^a^	7.4 ± 4.1 ^b^	24.5 ± 22.3 ^ab^
		Canopy	40.0 ± 5.5 ^a^	2.5 ± 2.7 ^b^	40.8 ± 3.8 ^a^

**Table 5:** Characteristics of microbial community in *P. antennata* for different clusters.

Cluster	Number of Taxa with Cut-Off Filter			Dominant Taxa (%)	
	Total	>0.1%	>1%	1st	2nd
1	427	27	7	*Caballeronia* (43.9)	*Paraburkholderia* (25.3)
2	171	22	7	*Paraburkholderia* (54.3)	*Caballeronia* (31.5)
3	314	25	7	*Paraburkholderia* (64.6)	*Caballeronia* (21.7)
Total	670	27	7	*Paraburkholderia* (48.1)	*Caballeronia* (32.4)

## References

[1] Dethlefsen L., McFall-Ngai M., Relman D.A. (2007). An ecological and evolutionary perspective on human–microbe mutualism and disease. Nature.

[2] Forsythe P., Sudo N., Dinan T., Taylor V.H., Bienenstock J. (2010). Mood and gut feelings. Brain Behav. Immun..

[3] Sekirov I., Russell S.L., Antunes L.C.M., Finlay B.B. (2010). Gut microbiota in health and disease. Physiol. Rev..

[4] Hooper L.V., Littman D.R., Macpherson A.J. (2012). Interactions between the microbiota and the immune system. Science.

[5] Amato K.R. (2013). Co-evolution in context: The importance of studying gut microbiomes in wild animals. Microbiome Sci. Med..

[6] Shapira M. (2016). Gut microbiotas and host evolution: Scaling up symbiosis. Trends Ecol. Evol. (Amst.).

[7] Tiede J., Scherber C., Mutschler J., McMahon K.D., Gratton C. (2017). Gut microbiomes of mobile predators vary with landscape context and species identity. Ecol. Evol..

[8] Teyssier A., Rouffaer L.O., Saleh Hudin N.S., Strubbe D., Matthysen E., Lens L., White J. (2018). Inside the guts of the city: Urban-induced alterations of the gut microbiota in a wild passerine. Sci. Total Environ..

[9] Savage A.M., Hackett B., Guénard B., Youngsteadt E.K., Dunn R.R. (2015). Fine-scale heterogeneity across Manhattan’s urban habitat mosaic is associated with variation in ant composition and richness. Insect Conserv. Divers..

[10] Engel P., Moran N.A. (2013). The gut microbiota of insects - diversity in structure and function. FEMS Microbiol. Rev..

[11] Schwab A., Dubois D., Fried P.M., Edwards P.J. (2002). Estimating the biodiversity of hay meadows in north-eastern Switzerland on the basis of vegetation structure. Agric. Ecosyst. Environ..

[12] Bröring U., Wiegleb G. (2005). Soil zoology II: Colonization, distribution, and abundance of terrestrial Heteroptera in open landscapes of former brown coal mining areas. Ecol. Eng..

[13] Frank T.H., Kunzle I.R. (2006). Effect of early succession in wildflower areas on bug assemblages (Insecta: Heteroptera). Eur. J. Entomol..

[14] Galle R., Torma A., Körmöczi L. (2010). Small-scale effect of habitat heterogeneity on invertebrate assemblages in sandy grasslands (Hungarian Great Plain). Pol. J. Ecol..

[15] Torma A., Varga C., Varga M. (2010). Spatial pattern of true bugs (Heteroptera) in heterogeneous grassland—preliminary results. Acta Phytopathol. Entomol. Hung..

[16] Torma A., Császár P. (2013). Species richness and composition patterns across trophic levels of true bugs (Heteroptera) in the agricultural landscape of the lower reach of the Tisza River Basin. J. Insect Conserv..

[17] Brändle M., Amarell U., Auge H., Klotz S., Brandl R. (2001). Plant and insect diversity along a pollution gradient: Understanding species richness across trophic levels. Biodivers. Conserv..

[18] Henry T.J. (1997). Phylogenetic analysis of family groups within the infraorder Pentatomomorpha (Hemiptera: Heteroptera), with emphasis on the Lygaeoidea. Ann. Entomol. Soc. Am..

[19] Ahn S.J. (2010). Hemiptera of Korea.

[20] Audsley M.D., Seleznev A., Joubert D.A., Woolfit M., O’Neill S.L., McGraw E.A. (2018). *Wolbachia* infection alters the relative abundance of resident bacteria in adult *Aedes aegypti* mosquitoes, but not larvae. Mol. Ecol..

[21] Kikuchi Y., Hosokawa T., Fukatsu T. (2011). An ancient but promiscuous host–symbiont association between *Burkholderia* gut symbionts and their heteropteran hosts. ISME J..

[22] Phillips J.N., Berlow M., Derryberry E.P. (2018). The effects of landscape urbanization on the gut microbiome: An exploration into the gut of urban and rural white-crowned sparrows. Front. Ecol. Evol..

[23] Crotti E., Balloi A., Hamdi C., Sansonno L., Marzorati M., Gonella E., Favia G., Cherif A., Bandi C., Alma A. (2012). Microbial symbionts: A resource for the management of insect-related problems. Microb. Biotechnol..

[24] Crotti E., Sansonno L., Prosdocimi E.M., Vacchini V., Hamdi C., Cherif A., Gonella E., Marzorati M., Balloi A. (2013). Microbial symbionts of honeybees: A promising tool to improve honeybee health. New Biotechnol..

[25] Berasategui A., Shukla S., Salem H., Kaltenpoth M. (2016). Potential applications of insect symbionts in biotechnology. Appl. Microbiol. Biotechnol..

[26] Kautz S., Rubin B.E., Russell J.A., Moreau C.S. (2013). Surveying the microbiome of ants: Comparing 454 Pyrosequencing with traditional methods to uncover bacterial diversity. Appl. Environ. Microbiol..

[27] Rubin B.E., Sanders J.G., Hampton-Marcell J., Owens S.M., Gilbert J.A., Moreau C.S. (2014). DNA extraction protocols cause differences in 16S rRNA amplicon sequencing efficiency but not in community profile composition or structure. Microbiol. Open.

[28] Fadrosh D.W., Ma B., Gajer P., Sengamalay N., Ott S., Brotman R.M., Ravel J. (2014). An improved dual-indexing approach for multiplexed 16S rRNA gene sequencing on the Illumina MiSeq platform. Microbiome.

[29] Yoon S.H., Ha S.M., Kwon S., Lim J., Kim Y., Seo H., Chun J. (2017). Introducing EzBioCloud: A taxonomically united database of 16S rRNA gene sequences and whole-genome assemblies. Int. J. Syst. Evol. Microbiol..

[30] Calle M.L. (2019). Statistical analysis of metagenomics data. Genom. Inform..

[31] USDA (United States Department of Agriculture) Forest Service Forest Inventory and Analysis National Program-Field Methods for Forest Health (Phase 3) Measurements ver 3.0. October 2005. https://www.fia.fs.fed.us/library/field-guides-methods-proc/index.php.

[32] Kőrösi Á., Batáry P., Orosz A., Rédei D., Báldi A. (2012). Effects of grazing, vegetation structure and landscape complexity on grassland leafhoppers (Hemiptera: Auchenorrhyncha) and true bugs (Hemiptera: Heteroptera) in Hungary. Insect Conserv. Divers..

[33] Rossetti M.R., Rösch V., Videla M., Tscharntke T., Batáry P. (2019). Insect and plant traits drive local and landscape effects on herbivory in grassland fragments. Ecosphere.

[34] Mielke P.W., Berry K.J., Johnson E.S. (1976). Multi-response permutation procedures for a priori classifications. Commun. Stat. Theor. Methods.

[35] Palavesam A., Guerrero F.D., Heekin A.M., Wang J., Dowd S.E., Sun Y., Foil L.D., Pérez de León A.A.P. (2012). Pyrosequencing-based analysis of the microbiome associated with the horn fly, *Haematobia irritans*. PLoS ONE.

[36] McCune B., Mefford M.J. (1999). PC-ORD: Multivariate analysis of ecological data. MjM software design. 4th Version for Windows; [User’s Guide].

[37] Niemelä J. (1999). Is there a need for a theory of urban ecology?. Urban Ecosyst..

[38] Meillère A., Brischoux F., Henry P.Y., Michaud B., Garcin R., Angelier F. (2017). Growing in a city: Consequences on body size and plumage quality in an urban dweller, the house sparrow (*Passer domesticus*). Lands. Urban. Plan..

[39] Lowe E.C., Threlfall C.G., Wilder S.M., Hochuli D.F. (2018). Environmental drivers of spider community composition at multiple scales along an urban gradient. Biodivers. Conserv..

[40] Sawana A., Adeolu M., Gupta R.S. (2014). Molecular signatures and phylogenomic analysis of the genus *Burkholderia*: Proposal for division of this genus into the emended genus *Burkholderia* containing pathogenic organisms and a new genus *Paraburkholderia* gen. nov. harboring environmental species. Front. Genet..

[41] Dobritsa A.P., Samadpour M. (2016). Transfer of eleven species of the genus *Burkholderia* to the genus *Paraburkholderia* and proposal of *Caballeronia* gen. nov. to accommodate twelve species of the genera *Burkholderia* and *Paraburkholderia*. Int. J. Syst. Evol. Microbiol..

[42] Rahman M., Sabir A.A., Mukta J.A., Khan M.M.A., Mohi-Ud-Din M., Miah M.G., Rahman M., Islam M.T. (2018). Plant probiotic bacteria Bacillus and *Paraburkholderia* improve growth, yield and content of antioxidants in strawberry fruit. Sci. Rep..

[43] Tago K., Okubo T., Itoh H., Kikuchi Y., Hori T., Sato Y., Nagayama A., Hayashi K., Ikeda S., Hayatsu M. (2015). Insecticide-degrading *Burkholderia* symbionts of the stinkbug naturally occupy various environments of sugarcane fields in a Southeast island of Japan. Microbes Environ..

[44] Kikuchi Y., Hayatsu M., Hosokawa T., Nagayama A., Tago K., Fukatsu T. (2012). Symbiont-mediated insecticide resistance. Proc. Natl. Acad. Sci. USA.

[45] Yun J.H., Roh S.W., Whon T.W., Jung M.J., Kim M.S., Park D.S., Yoon C., Nam Y.D., Kim Y.J., Choi J.H. (2014). Insect gut bacterial diversity determined by environmental habitat, diet, developmental stage, and phylogeny of host. Appl. Environ. Microbiol..

[46] Zhang Y.C., Cao W.J., Zhong L.R., Godfray H.C.J., Liu X.D. (2016). Host plant determines the population size of an obligate symbiont (Buchnera aphidicola) in aphids. Appl. Environ. Microbiol..

[47] Jones J.C., Fruciano C., Hildebrand F., Al Toufalilia H., Balfour N.J., Bork P., Engel P., Ratnieks F.L.W., Hughes W.O. (2018). Gut microbiota composition is associated with environmental landscape in honey bees. Ecol. Evol..

[48] Rajagopal T., Singam P., Kulandaivel S., Selvarani S., Sevarkodiyone S., Ponmanickam P. (2019). The red weaver ant, *Oecophylla smaragdina* as vectors of bacteria in urban environments. Int. J. Entomol. Res..

[49] Schneider M., Dorn A. (2001). Differential infectivity of two *Pseudomonas* species and the immune response in the milkweed bug, *Oncopeltus fasciatus* (Insecta: Hemiptera). J. Invertebr. Pathol..

